# Nutritional intake, environmental factors, and their impact on myopia prevalence in Korean children aged 5–12 years

**DOI:** 10.1186/s41043-024-00506-6

**Published:** 2024-01-29

**Authors:** Jeong-Mee Kim, Yean-Jung Choi

**Affiliations:** 1 Department of Visual Optics, Far East University, Eumseong, South Korea; 2 Department of Food and Nutrition, Sahmyook University, 815, Hwarang-ro, Nowon-gu, Seoul, 01795 South Korea

**Keywords:** Myopia, Dietary habits, Environmental factors, Children, Nutrients, Health

## Abstract

**Background:**

Myopia is a complex condition influenced by numerous factors, including genetic predisposition, environmental factors, and lifestyle choices. Although evidence indicates that certain dietary factors may influence the development of myopia, this relationship is still not completely understood and is a topic of ongoing research.

**Methods:**

This study analyzed the relationship between dietary habits, environmental factors, and the prevalence of myopia in a sample of 24,345 children aged 5–12 years from the seventh Korea National Health and Nutrition Examination Survey (KNHANES VII). The average daily intake of dietary nutrients associated with the refractive error status of the participants was analyzed using analysis of variance (GLM) and the Scheffe method for post-hoc comparison. Multiple logistic regression analysis was conducted between the participant’s refractive error status and daily dietary nutrient intake, while taking into consideration the age, sex, BMI, parental myopia, and near-work hours.

**Results:**

The risk of myopia increased with age, especially notable between ages 11 and 12, and was higher in children with both parents having myopia. Dietary factors played a crucial role; children with myopia had significantly lower intake of fat, omega-3 fatty acids, and retinol but higher intake of other nutrients compared to emmetropic and hyperopic counterparts. High consumption of carbohydrates, protein, phosphorus, iron, potassium, and sodium was associated with increased myopia risk. High sodium intake was particularly associated with a 2.05-fold increased myopia risk.

**Conclusions:**

This study highlights the significant role of diet and lifestyle choices in the development of myopia in children. Our findings suggest the importance of considering these specific factors in the management and prevention strategies for myopia, underscoring the need for targeted interventions in children's health and vision care.

## Background

The global prevalence of myopia is rapidly escalating, with East Asia being determined as one of the regions with the highest prevalence [1]. In fact, approximately 80–90% of school-aged children in this region have myopia [2]. South Korea, an East Asian nation, particularly reports a high prevalence of myopia of approximately 65% in children (aged 5–18 years) [3, 4]. Early onset of myopia has been associated with an increased risk of progression to high myopia [5, 6], which may lead to severe visual impairment owing to complications, such as retinal detachment, glaucoma, cataracts, and myopic macular degeneration, later in life [7–9]. Therefore, the high prevalence of myopia in children poses a mounting societal burden, warranting improved treatment and management strategies.

Numerous studies on myopia have indicated that the onset of myopia is influenced by both genetic and acquired factors, such as environmental conditions and nutritional status [10–13]. Currently, it is widely acknowledged that environmental factors, including near-work activity, outdoor light or time spent outdoors, have a substantial impact on the prevalence of myopia [14–16]. Moreover, a child's nutritional status significantly influences growth and development, including ocular development. Hence, diet has recently been recognized as a modifiable risk factor for myopia, potentially affecting its onset and progression. Several studies have investigated the association between nutrients and myopia, with varying results [13, 17, 18]. In a study on Chinese children aged 11–14 years, Ren et al. observed a correlation between sugary food consumption and myopia prevalence [17]. Conversely, Yin et al. found that dietary patterns rich in meats, seafood, dairy products, eggs, legumes, vegetables, fruits, grains, and potatoes were associated with a reduced risk of myopia among Chinese children aged 10–11 years (n = 7423) [13]. However, Li et al. found no association between specific nutrients or food groups and myopia in Singaporean children aged 9 years (n = 467) [18].

Several nutrients, such as omega-3 fatty acids, carotenoids, and antioxidant vitamins, are known for their protective roles in visual acuity, retinal function, and oxidative stress mitigation [19, 20]. As these nutrients can be obtained from the diet, it is worthwhile to explore the relationship between dietary nutrients and myopia. Hence, this study aimed to investigate the association between daily dietary nutrients and myopia in Korean children aged 5–12 years, leveraging data from the seventh Korea National Health and Nutrition Examination Survey (KNHANES VII) 2016 population-based dietary survey.

## Methods

### Data sources and study population

Data were obtained from the KNHANES VII, a cross-sectional population-based survey conducted by the Korea Centers for Disease Control and Prevention in 2016. This survey included health, dietary, and health examination questionnaires; thereafter, the raw data were analyzed.

**Fig. 1:**
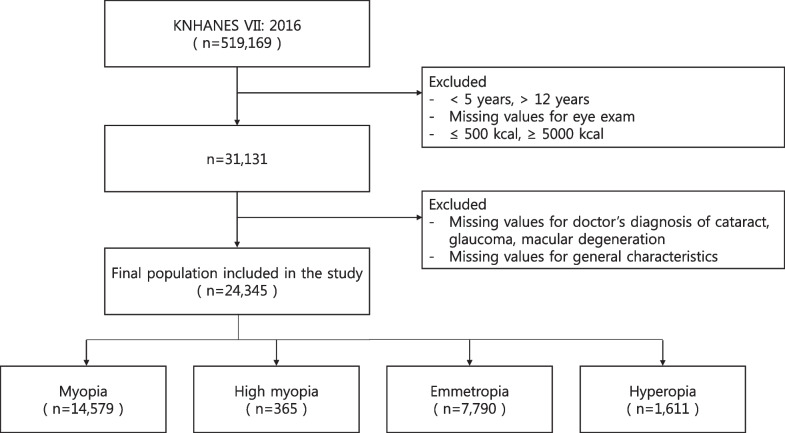
Flow chart of the selection process of the study participants

Of the 519,169 participants surveyed in KNHANES VII 2016, we focused on 31,131 individuals aged between 5 and 12 years after excluding data with missing values for refractive error and a total energy intake of less than 500 kcal/day or more than 5,000 kcal/day. Additionally, 640 children with a history of ophthalmic surgery for conditions such as strabismus or blepharoptosis were excluded, resulting in 30,491 children. The final sample comprised 24,345 individuals, after excluding those lacking data on variables relevant to general characteristics (Fig. 1).

In 2016, the KNHANES VII survey was conducted by the Korea Centers for Disease Control and Prevention. Review by the Institutional Review Board for Public Welfare was not required in accordance with the Bioethics Law. This survey was conducted in accordance with the ethical principles of the Declaration of Helsinki.

### Demographic and socio-economic data collection

Sociodemographic details and ocular history were collected using an interviewer-administered questionnaire. Body Mass Index (BMI) was calculated using the following formula: body weight (in kg) divided by height (in meters) squared. Household monthly income was categorized into quartiles, with 1 indicating the lowest income bracket and 4 indicating the highest income bracket. We also collected data on the presence of parental myopia and number of hours dedicated to near work per day. The participants’ living environments were classified as either urban or rural.

### Ocular examinations and dietary intake assessment

The refractive error in both eyes was measured using an auto-refractor keratometer (KR-8800; Topcon, Tokyo, Japan) without cycloplegia. The refractive error was subsequently calculated as the spherical equivalent (SE) of the sphere plus half of the cylinder based on the auto-refraction results. In this study, the SE of the right eye was used. The refractive error status was categorized according to the SE values as follows: myopia was defined as SE ≤ − 0.50 diopters (D), high myopia as SE ≤ − 6.00 D, hyperopia as SE ≥  + 0.75 D, and emmetropia as − 0.50 D < SE <  + 0.75 D.

The nutrition survey component of the KNHANES, which addresses dietary behaviors, food frequency, and food intake, was conducted via face-to-face interviews. Nutrient intake was determined using the dataset from the nutrition survey of the KNHANES. Twenty-four nutrients including carbohydrates, protein, fat, fiber, sodium, and vitamin C were considered in the nutrient intake survey. The food intake questionnaire was devised as an open-ended survey that allowed participants to report on various dishes and foods consumed using the individual 24-h recall method. Daily nutrient intake was calculated as the sum of nutrient intake from all food sources consumed by an individual during the day. The 2020 Dietary Reference Intakes for Koreans (KDRI) was used to assess whether nutrient intake was inadequate or excessive according to the age and sex.

The Nutrient Adequacy Ratio (NAR) represents a value that is set to 1 when the ratio of nutrient intake for a specific nutrient exceeds 1. Meanwhile, the Mean Adequacy Ratio (MAR) is the average of all NAR values and serves as an index indicating the overall quality of nutrient intake. The Index of Nutritional Quality (INQ) is determined by dividing the nutrient intake per 1000 kcal by the recommended nutrient intake for the same amount of calories. An INQ of 1 or above signifies that the nutrient is adequately consumed when the energy intake requirement is met. This metric is utilized to assess the nutritional balance of a meal.

### Statistical analysis

All the data were processed and analyzed using the SAS software (version 9.4; SAS Institute Inc., Cary, NC, USA). Each variable was analyzed using the complex sample design data analysis method, accounting for the clustering variables, stratification variables, and weights presented in the 7th KNHANES. Descriptive statistics, such as mean, frequency, and standard deviation, were calculated for age, refractive error (SE), and BMI. The participants were categorized into myopia (high myopia), emmetropia, and hyperopia groups. Categorical variables were analyzed using the chi-square test, and continuous variables were analyzed using the analysis of covariance test. Univariate logistic regression analysis was used to evaluate the association between the independent variables and myopia, with adjustments for age and sex. The average daily intake of dietary nutrients associated with the refractive error status of the participants was analyzed using analysis of variance (GLM) and the Scheffe method for post-hoc comparison. To account for multiple comparisons, we applied the Bonferroni correction method, ensuring rigorous statistical significance testing. A p-trend analysis was also conducted among the three groups (myopia, emmetropia, hyperopia) to identify trends in nutrient intake and refractive error associations. Finally, multiple logistic regression analysis was conducted between the participant's refractive error status and daily dietary nutrient intake, while taking into consideration the age, sex, BMI, parental myopia, and near-work hours. Statistical significance of all data analysis results was set at *p* < 0.05.

## Results

### Study population and general characteristics

Our study included 24,345 children aged 5–12 years, with 51.84% males and 48.16% females. The mean SE refractive error was − 2.37 (1.55) in children with myopia (61.38%), + 0.01 (0.29) in children with emmetropia (32.00%), and + 1.84 (1.04) in children with hyperopia (6.62%). We observed a significantly higher BMI in children with myopia than those without (*p* = 0.0315). Additionally, approximately 70% of the children had a parental history of myopia.

Parameters, such as age, sex, parental myopia, time spent on near-work activities, household income, and area of residence, exhibited statistically significant differences when stratified by refractive error status (*p* < 0.0001 for all). The general characteristics of the participants aged 5–12 years, categorized by refractive error status, are summarized in Table 1.

**Table 1:** Characteristics of participants aged 5–12 years according to the refractive error status in the right eye

	Total	Myopia (SE ≤ − 0.50 D)	Emmetropia (− 0.50 < SE < + 0.75 D)	Hyperopia (SE ≥ + 0.75 D)	*p*-value
Number	24,345	14,944 (61.38%)	7790 (32.00%)	1611 (6.62%)	
Age (years)					< .0001
Mean (SD)	9.00 (1.94)	9.58 (1.84)	8.17 (1.74)	7.60 (1.64)	
Gender, n (%)					< .0001
Male	12,620 (51.84%)	7723 (51.68%)	3789 (48.64%)	1108 (68.78%)	
Female	11,725 (48.16%)	7221 (48.32%)	4001 (51.36%)	503 (31.22%)	
Refractive error (SE) (D)					< .0001
Mean (SD)	− 1.33 (1.86)	− 2.37 (1.55)	+ 0.01 (0.29)	+ 1.84 (1.04)	
BMI (kg/m^2^)					0.0315
Mean (SD)	17.82 (3.23)	18.16 (3.18)	17.32 (3.22)	17.13 (3.23)	
Parental myopia, n (%)					< .0001
Father	4492 (18.45%)	2824 (18.90%)	1499 (19.24%)	169 (10.49%)	
Mother	5407 (22.21%)	3342 (22.36%)	1806 (23.18%)	259 (16.08%)	
Both parents	6804 (27.95%)	4676 (31.29%)	1850 (23.75%)	278 (17.26%)	
Neither of the parents	7642 (31.39%)	4102 (27.45%)	2635 (33.83%)	905 (56.18%)	
Near work, n (%)					< .0001
≤ 2 h/day	12,174 (50.01%)	7226 (48.35%)	4171 (53.54%)	777 (48.23%)	
3 h/day	5991 (24.61%)	4006 (26.81%)	1714 (22.00%)	271 (16.82%)	
≥ 4 h/day	6180 (25.39%)	3712 (24.84%)	1905 (24.45%)	563 (34.95%)	
Household income, n (%)					< .0001
Low	1277 (5.25%)	681 (4.56%)	523 (6.71%)	73 (4.53%)	
Middle	14,030 (57.63%)	8310 (55.61%)	4459 (57.24%)	1261 (78.27%)	
High	9038 (37.12%)	5953 (39.84%)	2808 (36.05%)	277 (17.19%)	
Residence, n (%)					< .0001
Urban	16,005 (65.74%)	9947 (66.56%)	5165 (66.30%)	893 (55.43%)	
Rural	8340 (34.26%)	4997 (33.44%)	2625 (33.70%)	718 (44.57%)	
Second hand smoking exposure, n (%)					< .0001
Yes	1073 (4.41%)	486 (3.25%)	514 (6.60%)	73 (4.53%)	
No	6306 (25.90%)	3511 (23.49%)	2205 (28.31%)	590 (36.62%)	
No one smokes indoors regularly at home	16,966 (69.69%)	10,947 (73.25%)	5,071 (65.10%)	948 (58.85%)	
Urine cotinine level (µg/mL)					< .0001
Mean (SD)	0.46 (0.68)	0.46 (0.81)	0.43 (0.40)	0.56 (0.41)	

*D* diopters, *SD* standard deviation, *SE* spherical equivalent, *BMI* body mass index

Table 2 illustrates the prevalence of refractive error according to the age group. Among the participating children aged 5–12 years, the largest proportion was represented by those with myopia (high myopia at 1.50%), accounting for 59.88% of the total participants. The prevalence of myopia increased with age. We also observed an increase in the prevalence of high myopia with age. The proportion of children with emmetropic and hyperopic eyes decreased with age.

**Table 2:** Prevalence of the refractive error status in the right eye by age group

Age group (years)	Myopia (SE ≤ − 0.50 D)	High myopia (SE ≤ − 6.00 D)	Emmetropia (− 0.50D < SE < + 0.75D)	Hyperopia (SE ≥ + 0.75 D)	*p*-value
	n (%)				
5–6	934 (30.50%)	0 (0%)	1540 (50.29%)	588 (19.20%)	< .0001
7–8	3530 (47.43%)	0 (0%)	3360 (45.14%)	553 (7.43%)	< .0001
9–10	4654 (67.39%)	56 (0.81%)	1841 (26.66%)	355 (5.14%)	< .0001
11–12	5461 (78.76%)	309 (4.46%)	1049 (15.13%)	115 (1.66%)	< .0001
Total	14,579 (59.88%)	365 (1.50%)	7790 (32.00%)	1611 (6.62%)	< .0001

Age group (years)	Myopia (SE) (D)	High myopia (SE) (D)	Emmetropia (SE) (D)	Hyperopia (SE) (D)	*p*-value
	LSMean (SE)				
5–6	− 0.77 (0.02)	–	+ 0.10 (0.02)	+ 2.16 (0.03)	< .0001
7–8	− 2.02 (0.01)	–	− 0.02 (0.01)	+ 1.40 (0.04)	< .0001
9–10	− 2.06 (0.02)	− 6.25 (0.16)	− 0.05 (0.03)	+ 1.63 (0.06)	< .0001
11–12	− 2.87 (0.02)	− 6.29 (0.07)	+ 0.04 (0.04)	+ 3.00 (0.12)	< .0001
Total	− 2.27 (0.01)	− 6.29 (0.06)	+ 0.01 (0.01)	+ 1.84 (0.03)	< .0001

*D* diopters, *SE* spherical equivalent, *SD* standard deviation

### Risk factors associated with myopia in children

In the model adjusted for age and gender, as outlined in Table 3, the risk of myopia was highest among children aged between 11 and 12 years at 11.54-fold (95% confidence interval [CI] 10.39–12.82; *p* < 0.0001). When both parents were myopic, the prevalence of myopia in the children was 2.04-fold higher (95% CI 1.89–2.20; *p* < 0.0001). Conversely, children residing in urban areas had the higher risk of myopia, at 1.09-fold (95% CI 1.02–1.15; *p* = 0.0066).

**Table 3:** Association of the risk factors in children with myopia aged 5–12 years using univariate logistic regression analysis

	Crude OR [95% CI]	*p*-value	Adjusted OR^1)^ [95% CI]	*p*-value
Gender				
Male	1		1	
Female	1.017 [0.965, 1.070]	0.5325	0.957 [0.904, 1.012]	0.1252
Age (years)				
5–6	1		1	
7–8	2.055 [1.880, 2.248]	< .0001	2.066 [1.888, 2.260]	< .0001
9–10	4.887 [4.457, 5.358]	< .0001	4.935 [4.495, 5.418]	< .0001
11–12	11.293 [10.225, 12.474]	< .0001	11.538 [10.385, 12.819]	< .0001
BMI (kg/m^2^)	1.093 [1.083, 1.102]	< .0001	0.988 [0.979, 0.997]	0.0130
Parental myopia				
Father	1.461 [1.355, 1.576]	0.1117	1.923 [1.770, 2.090]	< .0001
Mother	1.397 [1.301, 1.499]	0.8612	1.725 [1.597, 1.864]	0.0087
Both parents	1.896 [1.771, 2.030]	< .0001	2.038 [1.889, 2.197]	< .0001
Neither of the parents	1		1	
Near work				
≤ 2 h/day	1		1	
3 h/day	1.382 [1.295, 1.474]	< .0001	1.246 [1.160, 1.338]	< .0001
≥ 4 h/day	1.030 [0.968, 1.096]	< .0001	0.625 [0.582, 0.670]	< .0001
Household income				
Low	1		1	
Middle	1.271 [1.133, 1.426]	0.5292	1.365 [1.204, 1.549]	0.3384
High	1.689 [1.500, 1.901]	< .0001	1.733 [1.524, 1.971]	< .0001
Residence				
Urban	1.098 [1.041, 1.160]	0.0007	1.085 [1.023, 1.150]	0.0066
Rural	1		1	
SHS exposure				
Yes	0.455 [0.402, 0.515]	< .0001	0.299 [0.260, 0.343]	< .0001
No	0.691 [0.651, 0.733]	0.5645	0.635 [0.596, 0.677]	0.0008
No one smokes indoors regularly at home	1		1	
Urine cotinine (µg/mL)	1.006 [0.969, 1.045]	0.7489	0.941 [0.904, 0.980]	0.0032

*OR* odds ratio, *CI* confidence interval, *BMI* body mass index, *SHS* second hand smoking

^1)^Adjusted OR for age, gender, and BMI

Compared with the unadjusted odds ratio (OR), male and having middle household income were not significantly associated with the occurrence of myopia in children in the adjusted OR (*p* = 0.1252 and *p* = 0.3384, respectively). Factors, such as age, BMI, parental myopia, time spent on near-work activities, household income, and area of residence were associated with myopia in children (Table 3).

### Dietary nutrient intake and myopia

Table 4 presents the daily nutrient intake of the children classified by the refractive error status. In this study, among children aged 5–12 years, children with myopia had a significantly lower average daily intake of fat, omega-3 fatty acids, and retinol than their emmetropic and hyperopic counterparts. However, it was observed that the average daily intake of nutrients, excluding protein, was statistically significantly higher in children with Myopia compared to those with Emmetropia.

**Table 4:** Average daily intake of dietary nutrients in children aged 5–12 years according to the refractive error status

Nutrients	Myopia (n = 14,944)	Emmetropia (n = 7790)	Hyperopia (n = 1611)	*p*-value^1)^
Energy (kcal)	1967.11 (5.36)^a,2)^	1894.81 (7.44)^b^	1831.88 (16.18)^c^	< .0001
Carbohydrate (g)	298.04 (0.38)^a^	286.88 (0.52)^b^	293.69 (1.14)^c^	< .0001
Protein (g)	66.67 (0.13)^a^	66.79 (0.19)^a^	65.99 (0.40)^a^	0.1844
Fat (g)	51.05 (0.14)^a^	55.47 (0.20)^b^	52.46 (0.44)^c^	< .0001
Cholesterol (mg)	300.32 (1.66)^a^	278.19 (2.31)^b^	310.51 (5.03)^a^	< .0001
N-3 fatty acid (g)	1.30 (0.01)^a^	1.37 (0.01)^b^	1.36 (0.02)^ab^	< .0001
Calcium (mg)	470.03 (1.70)^a^	457.80 (2.36)^b^	529.23 (5.13)^c^	< .0001
Phosphorus (mg)	1015.24 (1.73)^a^	977.37 (2.41)^b^	1029.27 (5.24)^c^	< .0001
Iron (mg)	14.20 (0.05)^a^	13.19 (0.07)^b^	13.28 (0.16)^b^	< .0001
Sodium (mg)	2912.65 (8.82)^a^	2644.88 (12.24)^b^	2760.13 (26.64)^c^	< .0001
Potassium (mg)	2564.12 (6.59)^a^	2280.39 (9.15)^b^	2403.13 (19.91)^c^	< .0001
Vitamin A (μgRE)	415.33 (4.05)^a^	327.38 (5.63)^b^	381.79 (12.25)^c^	< .0001
β-carotene (μg)	3244.31 (47.39)^a^	2130.73 (65.78)^b^	2671.63 (143.19)^c^	< .0001
Retinol (μg)	144.97 (0.97)^a^	149.82 (1.34)^b^	159.15 (2.92)^c^	< .0001
Vitamin C (mg)	86.03 (0.71)^a^	69.33 (0.98)^b^	65.82 (2.13)^b^	< .0001
*Energy distribution*				
% Carbohydrate	62.99 (0.06)^a^	61.40 (0.09)^b^	62.31 (0.20)^c^	< .0001
% Protein	13.80 (0.03)^a^	13.85 (0.04)^a^	13.58 (0.08)^b^	< .0001
% Fat	23.21 (0.06)^a^	24.75 (0.08)^b^	24.11 (0.17)^c^	< .0001

^a,b,c^Same letters indicate a non-significant difference between the groups based on the Scheffe multiple comparison test

^1)^Different between groups at α = 0.05 by ANCOVA test adjusted for age, sex, BMI and energy(except energy)

^2)^Age, sex, bmi and energy-adjusted least squares means (LSmeans)

Children with hyperopia had significantly lower daily intake levels of energy, fat, and vitamin C compared to those with emmetropia (*p* < 0.0001). Conversely, their average daily consumption of cholesterol, calcium, phosphorus, sodium, potassium, beta-carotene, and retinol was significantly higher than that of emmetropic children (p < 0.0001). However, there were no statistically significant differences in the average daily intake of protein, cholesterol, and omega-3 fatty acids between children with myopia and hyperopia.

Children with myopia who had high consumption of carbohydrates and protein exhibited an increased risk of myopia by 1.32 and 1.57 times, respectively, compared to those in the low intake reference group. Additionally, higher intakes of phosphorus, iron, and potassium were associated with a significant increase in myopia risk, at 1.67, 1.56, and 1.65 times respectively. High sodium intake was linked to a 2.06 times greater risk of developing myopia (95% CI 1.95–2.19; *p* < 0.0001). Similarly, those with high vitamin C intake had a 1.85-fold increased risk of myopia compared to the group with low vitamin C intake (Table 5). On the other hand, for the emmetropic group, daily consumption of all nutrients, except for fat, appeared to have a protective effect against myopia development (data not shown).

**Table 5:** Odds ratio of the highly intake of dietary nutrients and myopia in children aged 5–12 years by the multivariable logistic regression analysis

Nutrients	Myopia			
	Crude OR [95% CI]	*p*-value	Adjusted OR^1)^ [95% CI]	*p*-value
Energy (kcal)	1.674 [1.589, 1.764]	< .0001	1.169 [1.103, 1.240]	< .0001
Carbohydrate (g)	1.711 [1.624, 1.802]	< .0001	1.317 [1.243, 1.395]	< .0001
Protein (g)	2.250 [2.134, 2.372]	< .0001	1.570 [1.479, 1.667]	< .0001
Fat (g)	1.565 [1.486, 1.649]	< .0001	1.203 [1.135, 1.275]	< .0001
N-3 fatty acid (g)	1.574 [1.494, 1.658]	< .0001	1.107 [1.045, 1.174]	0.0006
Cholesterol (mg)	1.549 [1.471, 1.632]	< .0001	1.164 [1.099, 1.233]	< .0001
Calcium (mg)	1.197 [1.137, 1.261]	< .0001	1.117 [1.054, 1.184]	0.0002
Phosphorus (mg)	2.024 [1.920, 2.133]	< .0001	1.666 [1.571, 1.768]	< .0001
Iron (mg)	2.246 [2.131, 2.368]	< .0001	1.561 [1.472, 1.655]	< .0001
Sodium (mg)	2.697 [2.556, 2.845]	< .0001	2.064 [1.947, 2.189]	< .0001
Potassium (mg)	2.077 [1.971, 2.189]	< .0001	1.648 [1.555, 1.747]	< .0001
Vitamin A (μgRE)	1.412 [1.340, 1.487]	< .0001	1.097 [1.035, 1.162]	0.0018
β-carotene (μg)	1.606 [1.525, 1.692]	< .0001	1.206 [1.138, 1.278]	< .0001
Retinol (μg)	1.031 [0.979, 1.086]	0.2468	1.069 [1.010, 1.133]	0.0217
Vitamin C (mg)	1.829 [1.736, 1.928]	< .0001	1.853 [1.746,1.966]	< .0001

*OR* odds ratio, *CI* confidence interval

^1)^Adjusted OR for age, sex, BMI, parental myopia, and near-work hours

We employed the NAR, MAR, and INQ metrics to assess the appropriateness of Korean nutritional intake standards for children aged 9–11 years. Table 6 highlights the variations in NAR and MAR values among subjects based on their refractive error status. Specifically, the children with myopia demonstrated favorable NAR and MAR metrics for most nutrients, with the exceptions being fiber, calcium, potassium, and vitamins A and C. A similar pattern was evident in the children with hyperopia; however, for vitamin C, the NAR was 0.48, indicating a significant deficiency in this nutrient. Furthermore, INQ differences among the three groups were examined, as detailed in Table 7. Notably, the children with hyperopia displayed increased INQ metrics for calcium and vitamin A, but these values remained below 1.

**Table 6:** NAR and MAR of the participants aged 9–11 years

Nutrients	Myopia (n = 7581)	Emmetropia (n = 2522)	Hyperopia (n = 470)	*p*-value^1)^
NAR				
Protein (g)^2)^	1.270 ± 0.003	1.229 ± 0.005	1.163 ± 0.013	< .0001
Fiber (g)	0.759 ± 0.004	0.728 ± 0.006	0.765 ± 0.015	0.0004
Calcium (mg)	0.709 ± 0.004	0.631 ± 0.006	0.826 ± 0.015	< .0001
Phosphorus (mg)	1.531 ± 0.003	1.391 ± 0.006	1.420 ± 0.014	< .0001
Iron (mg)	1.729 ± 0.010	1.791 ± 0.017	1.810 ± 0.040	0.0013
Sodium (mg)	2.456 ± 0.010	2.141 ± 0.018	2.037 ± 0.042	< .0001
Potassium (mg)	0.774 ± 0.003	0.624 ± 0.005	0.723 ± 0.012	< .0001
Vitamin A (μgRE)	0.585 ± 0.006	0.488 ± 0.011	0.775 ± 0.027	< .0001
Vitamin C (mg)	0.918 ± 0.011	0.694 ± 0.019	0.481 ± 0.046	< .0001
MAR	1.192 ± 0.003	1.080 ± 0.005	1.111 ± 0.013	< .0001

*NAR* nutrient adequacy ratio, *MAR* mean adequacy ratio

^1)^Different between groups at α = 0.05 by ANCOVA test adjusted for age, sex, BMI, and energy

^2)^Age and sex-adjusted least squares means (Lsmeans)

**Table 7:** INQ of the participants aged 9–11 years

Nutrients	Myopia (n = 7581)	Emmetropia (n = 2522)	Hyperopia (n = 470)	*p*-value^1)^
Protein (g)^2)^	1.149 ± 0.002	1.112 ± 0.004	1.065 ± 0.010	< .0001
Fiber (g)	0.687 ± 0.003	0.668 ± 0.005	0.718 ± 0.013	0.0002
Calcium (mg)	0.657 ± 0.003	0.588 ± 0.006	0.776 ± 0.014	< .0001
Phosphorus (mg)	1.409 ± 0.003	1.279 ± 0.005	1.310 ± 0.012	< .0001
Iron (mg)	1.577 ± 0.008	1.663 ± 0.014	1.706 ± 0.034	< .0001
Sodium (mg)	2.230 ± 0.009	1.989 ± 0.016	1.956 ± 0.037	< .0001
Potassium (mg)	0.716 ± 0.003	0.584 ± 0.005	0.670 ± 0.011	< .0001
Vitamin A (μgRE)	0.547 ± 0.007	0.436 ± 0.012	0.767 ± 0.029	< .0001
Vitamin C (mg)	0.840 ± 0.009	0.723 ± 0.016	0.425 ± 0.039	< .0001

*Inq* Index nutritional quality

^1)^Different between groups at α = 0.05 by ANCOVA test adjusted for age, sex, BMI, and energy

^2)^Age and sex-adjusted least squares means (Lsmeans)

## Discussion

The high prevalence of myopia among Korean children encouraged our study on the association between daily dietary nutrient intake and myopia development. From a nutritional perspective, our study unearthed a notable link between dietary habits and refractive error status. Children with myopia generally consumed a more balanced diet, with a few exceptions. This trend might hint at a conscious health-oriented approach by those maintaining or transitioning to a healthier diet based on their refractive error status.

This study found that myopic children generally had a higher dietary intake of most nutrients compared to their emmetropic and hyperopic counterparts. Notably, children with emmetropia showed significantly higher consumption of fat and omega-3 fatty acids. This pattern was also evident in energy distribution, with the emmetropic group having the highest proportion of fat intake. Conversely, children with hyperopia had the lowest vitamin C intake among the three groups. Furthermore, in terms of energy distribution, the group with myopia had the highest carbohydrate intake. These observations suggest a potential link between dietary habits and refractive error status.

Several previous studies have explored the association between diet and myopia [17, 21, 22], although the specific nutrients and age groups examined varied. A study by Ren et al. found a positive correlation between myopia prevalence and the consumption of sugar-containing processed foods, such as cakes, canned fruit, sweet, chocolate, and ice cream, among Chinese children aged 11–14 years. [17] Similarly, Liu et al. reported that consuming over 50% of whole grains was an independent protective factor against myopia in a cross-sectional epidemiological study of Chinese children aged 6–12 years [21]. Another study suggested that the consumption of refined carbohydrates might be associated with myopia in French children aged 4–18 years, with girls having a higher prevalence of myopia than boys [22]. Although our study did not distinguish between whole grains and refined carbohydrates, considering that the typical Korean diet consists of white rice, we found that the intake of refined carbohydrates by children exceeded the recommended daily carbohydrate intake as per the KDRI (according to The Korean Nutrition Society & Ministry of Health and Welfare, 2020). Our results also showed that Korean children with myopia had a higher average daily carbohydrate intake compared to those without myopia.

In contrast, a previous study by Edwards [23], which compared the dietary intake of 24 children with myopia aged 7–10 years in Hong Kong with that of 68 children without myopia 10-years, found that the children with myopia had lower intake of energy, protein, fat, phosphorus, iron, and cholesterol. However, our study found that the daily dietary intake of these nutrients was higher in the children with myopia. These conflicting results may be attributed to variations in the study design, sample size, dietary assessment methods, and population characteristics.

In a cross-sectional study of 851 Chinese schoolchildren aged 12–13 years from the Singapore Cohort Study of Risk Factors for Myopia (SCORM) conducted by Lim et al. [24] higher intake of saturated fat and cholesterol were associated with increased axial length but not myopia. Contrary to our findings, in a cohort study of 467 Singaporean children aged 9 years in the Growing Up in Singapore Towards Healthy Outcome (GUSTO) cohort by Li et al. [18], none of the 13 nutrients or food groups were associated with incident myopia or axial length.

Recent studies employing Mendelian randomization (MR) analysis have explored the causal links between specific nutrients or dietary patterns and myopia development [25, 26]. One study using MR examined the impact of genetically determined 25(OH)D levels on myopia severity. This study's MR estimates found no direct correlation between vitamin D levels and myopic refractive error (RE), indicating that individuals genetically predisposed to lower 25(OH)D levels were not more myopic than anticipated [25]. Deng et al. conducted MR analysis with extensive genome-wide association study (GWAS) data from the Social Science Genetic Association Consortium (SSGAC) to assess the effects of carbohydrate, protein, fat, and sugar intake on myopia. Their results indicated a positive correlation between protein and fat intake and increased risk of myopia, whereas carbohydrate and sugar intake showed no significant association with myopia risk [26]. While MR studies contribute to understanding the overall impact of various factors on outcomes and potential mediating influences, they do not conclusively establish direct causality [27, 28]. In our study involving children aged 5–12 years, the prevalence of myopia was the highest (61.38%), followed by emmetropia (32.00%) and hyperopia (6.62%). With advancing age, the prevalence of myopia increased, whereas the proportion of children with emmetropia and hyperopia, which is consistent with the findings of other studies [13, 29]. Notably, children with emmetropia with a refractive error of − 0.50 D < SE <  + 0.75 D are possibly in the pre-myopic stage and have a high likelihood of developing myopia in the future. This could be attributed to the increased educational exposure of early school-aged children, which correlates with a heightened risk of myopia. Countries with a higher prevalence of myopia often have more rigorous educational expectations for children, leading to earlier and more intensive tutoring, a trend from which Korean children are not exempt [30].

With societal progress, numerous environmental factors have evolved simultaneously, including education level, family income, population density, and living conditions, such as housing type, diet, and lifestyle. These factors reportedly correlate with the prevalence of myopia [31]. Our results also highlight that factors such as residential area, economic status, diet, and near-work activity are associated with myopia in children. Several studies have suggested that increased outdoor time and reduced near-work activities, including time spent using digital devices, may protect against the progression of myopia in children [32–34].

It has been hypothesized that nutrients involved in ocular growth, retinal function, and maintenance of eye health could potentially affect the development of myopia [35–38]; however, the exact mechanisms remain unclear. Current research on the relationship between nutrition and myopia development is ongoing, with several emerging hypotheses [13, 22, 39, 40]. Firstly, deficiencies in vitamins A, C, D, and B complex might impact collagen synthesis and scleral growth, potentially leading to a weaker sclera that is more prone to elongation and myopia. Secondly, a diet high in processed foods, refined carbohydrates, and sugary drinks could contribute to inflammation and oxidative stress, affecting scleral health and growth. Thirdly, nutritional factors during critical developmental stages like childhood and adolescence may have a substantial impact on the development of myopia. Lastly, chronic low-grade inflammation associated with diets high in fat and sugar could influence myopia through various mechanisms. Based on these insights, we propose a potential link between diet and the development of myopia. Although evidence indicates that certain dietary factors may influence the development of myopia, this relationship is still not completely understood and is a topic of ongoing research. Myopia is a complex condition influenced by numerous factors, including genetic predisposition, environmental factors, and lifestyle choices. [11, 31, 41]

The major advantage of our study was the use of a large population-based sample representative of the general South Korean child population. Although some studies have examined a limited range of specific nutrients, such as omega-3 fatty acids and sodium, our study investigated a wider range of nutrients, including both macronutrients and micronutrients, through daily dietary intake [19, 42].

However, this study had several limitations that should be acknowledged. First, the inability to perform cycloplegic refraction owing to the time constraints of the comprehensive KNHANES population-based examination might have led to an overestimation of myopia prevalence in children. The results of this study can be compared to some extent with other population-based studies that used autorefraction without ophthalmoplegia to evaluate refractive error [43–48]. However, numerous studies, including Hashemi et al. [49], have shown that accommodation in children can affect the accuracy of refractive error measurements, thus potentially skewing our results. We acknowledge that autorefraction without cycloplegia has been found to be less accurate for determining true refractive error, particularly in young children who have higher accommodative responses [50]. We agree that cycloplegic refraction, as suggested by multiple studies [49, 51–53], would yield more reliable and precise measurements. Unfortunately, due to time constraints and logistical considerations, we were unable to perform comprehensive eye exams, including cycloplegic refraction. In future research, we aim to incorporate comprehensive eye exams to improve the accuracy of our findings. Second, our study relied on self-reported dietary recall for data on nutrient intake, which could introduce recall bias or misreporting [54–58]. To mitigate this issue, we used standardized questionnaires and food recall methods to collect dietary data, though we recognize that this approach is not without its flaws. Future studies using more objective measures of dietary intake, such as biochemical assays or weighed food records, could provide more accurate results. Additionally, as recommended by prior research on multiple comparison tests [59–62], we also applied a Bonferroni correction to adjust the p-values in our statistical tests, mitigating potential inaccuracies that could result from large sample sizes. The corrected values continue to show significant associations between dietary nutrient intake and myopia, validating the original results to some extent. Third, the cross-sectional design limits our ability to establish causal relationships between nutrient intake and myopia, echoing limitations found in similar studies. Future research, especially longitudinal studies and randomized controlled trials, would be invaluable in corroborating these findings and establishing causality. Fourth, although the primary focus of our study was on dietary nutrient intake, we acknowledge that genetic predisposition plays a significant role in the development and progression of myopia. Studies have indeed identified several gene mutations associated with myopia [63–68]. However, our current study design did not include genetic profiling, so we were unable to account for this variable. Future studies could aim to incorporate genetic data to better understand the interplay between genetics and dietary intake in affecting myopia prevalence. Fifth, while acknowledging the significance of outdoor time in myopia research, we admit its absence is a limitation in our current analysis. Future studies would benefit from including this variable, offering a more complete understanding of myopia development in infants and young children. Sixth, while our study focused on children aged 5–12 years, it would certainly be valuable to consider adolescents as well. Puberty is a time of rapid physical change, including eye development [69, 70], and dietary needs also evolve during this period. Expanding the age range could provide insights into how the association between diet and myopia may change with age, especially given that adolescence is another critical period for myopia progression. However, doing so would also require controlling for other factors that become more variable in adolescence, such as hormonal changes and lifestyle differences, which our current study was not designed to accommodate. Lastly, while our study focused exclusively on Korean children, there are studies that suggest that diet may have varying impacts on myopia prevalence in different racial or ethnic groups [71–73]. For example, western diets high in processed foods have been linked to myopia, but the exact relationship and its generalizability across different racial backgrounds are still a subject of ongoing research. Therefore, while our study provides valuable insights, these limitations must be considered when interpreting the results.

## Conclusion

In our study, we observed that Korean children with and without myopia consume enough calories to meet their daily requirements. While it was noted that Korean children aged 5–12 years with myopia tended to have a higher overall intake of most nutrients compared to their non-myopic counterparts, this observation should not be interpreted as implying that nutritional deprivation is protective against myopia. Instead, our findings suggest that the relationship between dietary nutrient intake and myopia is complex and multifaceted. It is important to consider that while certain dietary patterns may be associated with myopia, they do not necessarily indicate a causal relationship. Our study highlights the need for further research to explore the effectiveness of dietary interventions in preventing or managing myopia in this population. Future prospective studies with larger sample sizes and longitudinal designs are needed to validate our findings and elucidate the specific mechanisms underlying the association between nutrient intake and myopia in Korean children.

## Data Availability

All the data files are available from the Korea Centers for Disease Control and Prevention database at https://knhanes.kdca.go.kr/knhanes/sub03/sub03_02_05.do. Individuals, including international researchers who sign up for membership, can access raw data from this website. However, the data-access process and user manual are written in Korean.

## Abbreviations

KNHANES: Korea National Health and Nutrition Examination Survey
BMI: Body mass index
SE: Refractive error
KDRI: Dietary reference intakes for Koreans
NAR: Nutrient adequacy ratio
MAR: Mean adequacy ratio
INQ: Index of nutritional quality
OR: Odds ratio
